# Endoplasmic Reticulum Stress, Unfolded Protein Response and Altered T Cell Differentiation in Necrotizing Enterocolitis 

**DOI:** 10.1371/journal.pone.0078491

**Published:** 2013-10-23

**Authors:** Peng Lu, Marie-Chantal Struijs, Jiaping Mei, Janneke Witte-Bouma, Anita M. Korteland-van Male, Adrianus C. J. M. de Bruijn, Johannes B. van Goudoever, Ingrid B. Renes

**Affiliations:** 1 Division of Neonatology, Department of Pediatrics, Erasmus MC-Sophia, Rotterdam, the Netherlands; 2 Department of Pediatrics, Emma Children’s Hospital - AMC, Amsterdam, The Netherlands; 3 Department of Pediatric Surgery, Erasmus MC-Sophia, Rotterdam, the Netherlands; 4 Neonatal Intensive Care Unit, Shenzhen Maternal and Child Healthcare Hospital, Affiliated Hospital of Southern Medical University, Shenzhen, Guangdong, China; 5 Department of Pediatrics, VU University Medical Center, Amsterdam, The Netherlands; New York University, United States of America

## Abstract

**Background:**

Endoplasmic reticulum (ER) stress and activation of the unfolded protein response (UPR) play important roles in chronic intestinal inflammation. Necrotizing enterocolitis (NEC) is the most common gastrointestinal emergency in preterm infants and is characterized by acute intestinal inflammation and necrosis. The objective of the study is to investigate the role of ER stress and the UPR in NEC patients.

**Methods:**

Ileal tissues from NEC and control patients were obtained during surgical resection and/or at stoma closure. Splicing of *XBP1* was detected using PCR, and gene expression was quantified using qPCR and Western blot.

**Results:**

Splicing of *XBP1* was only detected in a subset of acute NEC (A-NEC) patients, and not in NEC patients who had undergone reanastomosis (R-NEC). The other ER stress and the UPR pathways, PERK and ATF6, were not activated in NEC patients. A-NEC patients showing *XBP1* splicing (A-NEC-XBP1s) had increased mucosal expression of *GRP78*, *CHOP*, *IL6* and *IL8*. Similar results were obtained by inducing ER stress and the UPR *in* *vitro*. A-NEC-XBP1s patients showed altered T cell differentiation indicated by decreased mucosal expression of *RORC, IL17A* and *FOXP3*. A-NEC-XBP1s patients additionally showed more severe morphological damage and a worse surgical outcome. Compared with A-NEC patients, R-NEC patients showed lower mucosal *IL6* and *IL8* expression and higher mucosal *FOXP3* expression.

**Conclusions:**

XBP1 splicing, ER stress and the UPR in NEC are associated with increased *IL6* and *IL8* expression levels, altered T cell differentiation and severe epithelial injury.

## Introduction

Endoplasmic reticulum (ER) stress-related inflammation is involved in the pathogenesis of various chronic inflammatory diseases, including inflammatory bowel disease [1,2]. In the ER, secretory and transmembrane proteins are folded into their native conformation, and proper protein conformation needs the assistance of molecular chaperones such as 78 kDa glucose-regulated protein (GRP78). As such, highly secretory cells, like Paneth cells, have high basal levels of the molecular chaperone GRP78 to maintain homeostasis of protein folding in the ER [1,3]. When misfolded or unfolded proteins accumulate in the ER, ER stress occurs. To restore ER homeostasis, mammalian cells activate a process called unfolded protein response (UPR), which is marked by induction of a number of UPR-related genes including GRP78 and C/EBP homologous protein (CHOP). There are at least three ER stress sensors on the ER membrane, which are inositol-requiring transmembrane kinase-endoribonuclease-1 (IRE1), pancreatic ER kinase (PERK), and activated transcription factor 6 (ATF6) (Figure S1). 

Growing evidence shows that ER stress and the UPR play crucial roles in intestinal homeostasis and inflammation. In the colon and small intestine of patients with inflammatory bowel disease, ER stress and the UPR go hand in hand with increased GRP78 expression [4] and spliced X-box binding protein 1 (*XBP1s*) mRNA [5,6]. Furthermore, multiple single nucleotide polymorphisms of X-box binding protein 1 (XBP1) were found to be associated with inflammatory bowel disease in independent whole genome analysis studies [7–9]. However, ER stress and the UPR have not been studied in necrotizing enterocolitis (NEC), an acute intestinal inflammatory disease in premature infants. 

NEC is the most common gastrointestinal emergency in the neonatal intensive care unit, with high morbidity and mortality rates in very low birth weight infants [10]. NEC is most common in the ileum, and is characterized by inflammation and coagulation necrosis [11]. Current treatment strategies for NEC are antibiotic administration and, if required, eventually intestinal surgery to remove the affected part of the intestine. Infants, who underwent such surgery and survive, often experience severe long-term complications, such as intestinal strictures, recurrent sepsis, short bowel syndrome, growth impairment, and poor neurodevelopmental outcome [10]. These adverse sequelae underscore the importance of defining the pathophysiology of NEC to develop new strategies for treatment and prevention. 

In this study, we aimed to study ER stress and the UPR in the ileum of the NEC patients. Our data show that ER stress and the UPR occurred only in a subset of acute NEC patients but not in patients who recovered from NEC. Moreover, ER stress and the UPR correlated with poor surgical outcome, increased interleukin 6 (IL6) and *interleukin 6* (*IL8*) expression, and altered T cell differentiation. 

## Methods

### Study population and sample collection

This study was conducted with the approval of the 'Central Committee on Research involving Human Subjects', Hague, the Netherlands, and the written informed consents were obtained from the guardians on the behalf of the infant participants involved in the study. Infants who underwent bowel resection at the neonatal intensive care unit at Sophia Children’s Hospital between November 2003 and September 2010 were recruited. Fresh ileal tissue samples were collected during surgery, and fixed in 4 % paraformaldehyde in phosphate buffered saline or snap-frozen in liquid nitrogen immediately. Diagnosis and staging of NEC were based on Bell’s criteria [12], and diagnosis was confirmed during surgery and by histopathological evaluation of resected intestinal tissue. Preterm patients who underwent bowel resection for stage III acute NEC were referred to as A-NEC, and NEC patients who underwent reanastomosis (stoma closure) were referred to as R-NEC. Patients who underwent resection for diseases other than NEC were included as control patients. Control patients for A-NEC (A-CTRL) only contained preterm patients, and control patients for R-NEC (R-CTRL) contained patients who were born prematurely or at term. The corrected gestational ages during surgery of A-CTRL and A-NEC patients and R-CTRL and R-NEC patients were matched. The patient demographics and reasons for surgery on control patients are shown in Table 1 and Table 2, respectively. 

**Table 1:** Patient demographics.

Demographics	A-CTRL n=5	A-NEC n=12	R-CTRL n=10	R-NEC n=13
Male	3 (60.0%)	10 (83.3%)	6 (60.0%)	10 (76.9%)
Birth weight (gram)	905 (760, 1935)	1175 (785, 1526)	2323 (1400, 3411)^$^	950 (858, 1548)^$^
Gestational age at birth (weeks)	26.9 (26.8, 33.9)	29.5 (27.4, 31.1)	34.8 (28.5, 39.1)	29.4 (26.9, 31.2)
Postnatal age at surgery (days)	8.0 (1.0, 24.5)	10.5 (5.5, 19.8)^#^	43.5 (0.8, 83.0)^$^	78.0 (52.0, 120.5)^$,#^
Gestational age at surgery (weeks)	31.0 (28.8, 34.1)	31.0 (28.6, 32.9)^#^	40.6 (38.6, 42.1)	42.1 (36.0, 45.2)^#^
Survival (1 year after surgery)	5 (100.0%)	6 (50.0%)^#^	10 (100.0%)	12 (92.3%)^#^

A-NEC: preterm patients who underwent bowel resection for stage III acute NEC.

A-CTRL: control patients for A-NEC, premature patients who underwent resection for developmental defects or diseases other than NEC.

R-NEC: NEC patients who underwent another surgical procedure when eligible for reanastomosis (stoma closure) after full recovery.

R-CTRL: control patients for R-NEC, corrected gestational age matched both preterm and term patients who underwent resection for developmental defects or diseases other than NEC.

Data provided are numbers (percentage) or medians (interquartile range).

^$^ *P* < .05 for comparisons between R-CTRL and R-NEC.

# *P* < .05 for comparisons between A-NEC and R-NEC.

**Table 2:** Reasons for surgery on control patients.

Reason for surgery	n
A-CTRL	
Ileum perforation after incarcerated hernia	1
Isolated perforation	1
Milk curd syndrome	1
Meconium ileus and Meckels' diverticulum	1
Gastroschizis	1*
R-CTRL	
Ileum perforation after incarcerated hernia	1
Perforation with gastroschizis	1
Milk curd syndrome	1
Meckels' diverticulum	2
Meconium ileus and perforation	1
Midgut volvulus	1
Gastroschizis	1
Atresia	2

* *XBP1s* detected.

### Cell Culture and Treatment

The human monocytic cell line THP-1 (derived from the peripheral blood of a 1 year old human male) was purchased from ATCC, and the cells were cultured in Dulbecco’s modified Eagle’s minimal essential medium supplemented with 10 % fetal bovine serum, 50 μg/ml streptomycin and 50 U/ml penicillin. The fetal human intestinal epithelial cell line HIEC (a kind gift from Prof. Jean-François Beaulieu) was cultured in Opti-MEM I GlutaMAX medium supplemented with 5 % fetal bovine serum, 0.01 M HEPES and 5 ng/ml epidermal growth factor [13]. Both cell lines were cultured in a 37 °C incubator with 5 % CO_2_. To induce ER stress and the UPR, cells were treated with 5 μg/ml of tunicamycin (TM, purchased from Sigma-Aldrich) for 6 hours, and cells treated with dimethyl sulfoxide were used as control. 

### RNA isolation and complementary DNA synthesis

Total RNA from snap-frozen ileal tissue was isolated using RNeasy midi kit (Qiagen, Venlo, the Netherlands), and total RNA from THP-1 cells and HIEC cells was isolated using the NucleoSpin RNA II kit (Macherey-Nagel, Düren, Germany). The quality of the RNA samples was analyzed by Bioanalyzer (Agilent Technologies), and only RNA samples with an RNA integrity number greater than 7 were used for further analysis. Complementary DNA was synthesized from 1.5 μg RNA using M-MLV reverse transcriptase (Promega, Leiden, the Netherlands). 

### *XBP1s* detection

Complementary DNA was amplified using *XBP1* forward primer (5’-GGA GTT AAG ACA GCG CTT GGG GA-3’) and *XBP1* reverse primer (5’-TGT TCT GGA GGG GTG ACA ACT GGG-3’) to generate cDNA products encompassing the IRE1 cleavage sites [8]. The unspliced X-box binding protein 1 (*XBP1u*) and *XBP1s* mRNAs generate 164-bp and 138-bp PCR products, respectively. These fragments were resolved on 2 % agarose gel to distinguish the PCR products of *XBP1u* and *XBP1s*. 

### Quantitative PCR (qPCR)

Relative mRNA expression was determined using DyNAmo HS SYBR Green qPCR Kit (Finnzymes, Vantaa, Finland) on the ABI prism 7900HT Fast Real-Time PCR system (PE Applied Biosystems, Foster City, CA, USA), and was normalized against the mRNA expression levels of glyceraldehyde 3-phosphate dehydrogenase (*GAPDH*) or cluster of differentiation 4 (CD4) as described previously [14]. All qPCR primer sequences are given in Table 3. 

**Table 3:** QPCR primers used in this study.

Gene	Forward Primer (5’ to 3’)	Reverse Primer (5’ to 3’)
*GAPDH*	GTCGGAGTCAACGGATT	AAGCTTCCCGTTCTCAG
*ATF4*	AGCACCGCAACATGAC	CCACCTCCAGGTAATCATC
*GADD34*	TCCTCTGGCAATCCCCCATA	GGAACTGCTGGTTTTCAGCC
*PDIA4*	TCCCATTCCTGTTGCCAAGAT	GCCCTCGTAGTCTACAGCCT
*GRP78*	GCAGCAGGACATCAAGTT	TCAGCGGTTTCTTTCATT
*CHOP*	TTCGGGACACTGTCCA	CAGCCAAGCCAGAGAAG
*IL6*	CAGCCACTCACCTCTTCA	TTTGCTGCTTTCACACAT
*IL8*	TGCCAAGGAGTGCTAAAG	CAGCCCTCTTCAAAAACTT
*CD4*	GCCTCCTGCTTTTCATT	CTGGCAGGTCTTCTTCTC
*CD45*	TATGTTGTCAAGCTAAGGC	ATTCACTTCTGTTTCTCCAA
*TBX21*	GCCAGGAAGTTTCATTTG	CGGGGCTGGTACTTATG
*GATA3*	CACGGTGCAGAGGTAC	AAGGGGCTGAGATTCC
*RORC*	CCCTGCTGAGAAGGAC	AGGGATCACTTCAATTTGT
*IL17A*	AAAGGTCCTCAGATTACTAC	ATGGGGACAGAGTTCA
*FOXP3*	GACCCCCTTTCACCTAC	TGGCAGGATGGTTTCT
*CTLA4*	TCTCCTGTTTTTTCTTCTCTT	CCGTGCAGATGGAATC

### Western blot

Briefly, 20 µg of homogenates from snap-frozen tissue samples were separated on SDS-PAGE and subjected to Western blot. The blots were probed with antibodies against GRP78 (Santa Cruz Biotechnology, CA, USA), CHOP (Abnova, Heidelberg, Germany) and beta ACTIN (Abcam, Cambridge, UK). Signal was detected using IRDye 800CW conjugated secondary antibodies on the Odyssey Imager (Li-cor, Westburg, Leusden, the Netherlands). 

### Histology, immunohistochemistry (IHC) and immunofluorescence (IF)

Paraformaldehyde fixed ileal tissues were embedded in paraffin, and 4-μm-thick sections were stained with hematoxylin and eosin to study the ileal morphology. For IHC and IF, antigen unmasking was carried out by heating the sections for 20 minutes in 0.01 M sodium citrate (pH 6.0) at 100 °C [15]. Antibodies against GRP78 (Santa Cruz Biotechnology, CA, USA), human alpha defensin 5 (HD5, HyCult Biotechnology, Uden, the Netherlands), and Forkhead box P3 (FOXP3, eBioscience, San Diego, CA, USA) were used in combination with the Vectastain Elite ABC kit (Vector Laboratories, Burlingame, CA, USA) and 3,3’-diaminobenzidine as staining reagent for IHC, or in combination with DyLight 594 conjugated secondary antibody and DyLight 488 conjugated secondary antibody (Jackson ImmunoResearch, West Grove, PA, USA) for IF. 

### Statistical analysis

Data were presented as the total numbers (percentage), medians (interquartile range), or mean ± SEM. The comparisons were made by Fisher's exact test, Mann-Whitney test, or Kruskal-Wallis test followed by Dunns comparison. The data were considered statistically significant at *P* < .05. 

## Results

### ER stress and the UPR in NEC patients

**Figure 1:**
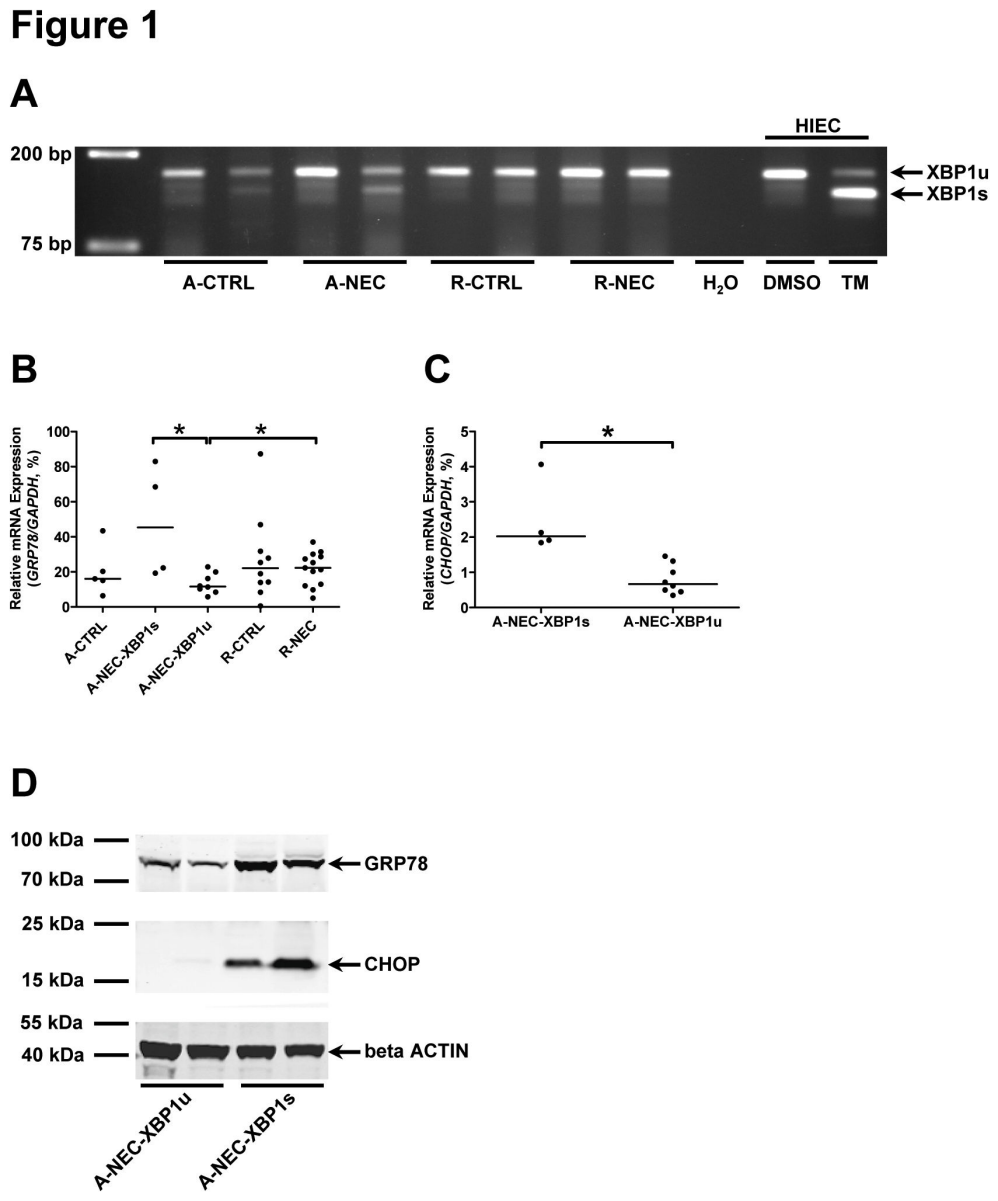
Occurrence of ER stress and the UPR in a subset of A-NEC patients. (A) Splicing of *XBP1* mRNA was checked in all patients, and representative PCR products of *XBP1u* and *XBP1s* are shown using DNA electrophoresis. Dimethyl sulfoxide (DMSO)-treated HIEC cells were used as negative control for *XBP1s*, and TM-treated HIEC cells were used as positive control for *XBP1s*. Mucosal mRNA expression levels of *GRP78* (B) and *CHOP* (C) in the ileum of patients were quantified using qPCR and normalized to *GAPDH* mRNA levels. Asterisks indicate statistical significant differences between indicated groups. (D) The Western blot result shows the representative protein expression of GRP78 and CHOP in A-NEC-XBP1u and A-NEC-XBP1s patients, and beta ACTIN was used as loading control.

Firstly, we analyzed splicing of *XBP1* mRNA to determine the activation of the IRE1 pathway, 1 of the 3 ER stress sensors and a hallmark of the UPR. There was no *XBP1s* detected in R-CTRL (n=10) and R-NEC (n=13) patients, and *XBP1s* was only detected in 1 out of 5 A-CTRL patients (20%) and in 4 out of 12 A-NEC (33%) patients (Figure 1A and Table 2). Based on the occurrence of *XBP1s*, A-NEC patients were further divided into two subsets, A-NEC patients in whom *XBP1s* was not detected, i.e., unspliced, (A-NEC-XBP1u) and A-NEC patients in whom the *XBP1s* was detected (A-NEC-XBP1s). The patient demographics of the two subsets of A-NEC patients are shown in Table 4. 

**Table 4:** A-NEC patient demographics.

Demographics	A-NEC-XBP1u n=8	A-NEC-XBP1s n=4	*p* Value
Male	6 (75.0%)	4 (100.0%)	0.5152
Birth weight (gram)	1001 (781, 1190)	1501 (926, 1909)	0.1481
Gestational age at birth (weeks)	27.8 (26.0, 29.7)	30.8 (29.9, 32.2)	0.0485
Postnatal age at surgery (days)	11.5 (7.8, 19.8)	7.0 (4.3, 30.8)	0.5697
Gestational age at surgery (weeks)	29.4 (27.8, 32.1)	32.6 (31.4, 34.9)	0.0727
Survival (1 year after surgery)	5 (62.5%)	1 (25.0%)	0.5455

A-NEC-XBP1u: acute NEC without *XBP1* splicing.

A-NEC-XBP1s: acute NEC with *XBP1* splicing.

Data provided are numbers (percentage) or medians (interquartile range).

Secondly, mRNA expression levels of activated transcription factor 4 (ATF4) [16] and growth arrest and DNA damage-inducible protein 34 (*GADD34*) [17] were analyzed to determine whether the PERK pathway, the second pathway sensing ER stress and the UPR, was activated. Neither increased *ATF4* nor *GADD34* mRNA expression was observed in any of the patient groups, and no significant differences in *ATF4* and *GADD34* mRNA expression levels were detected between A-NEC-XBP1s patients and A-NEC-XBP1u patients (Figure S2A and S2B). 

Thirdly, to investigate the activation of the ATF6, the third sensor of ER stress and the UPR pathway, mRNA expression of protein disulfide isomerase family A member 4 (*PDIA4*) was quantified [6]. Similar to *ATF4* and *GADD34*, no significant differences in *PDIA4* mRNA expression levels were detected among the patient groups, and the *PDIA4* mRNA expression in A-NEC-XBP1s patients was comparable to those in A-NEC-XBP1u patients (Figure S2C). 

Finally, expression levels of GRP78 and CHOP, general markers of ER stress and the UPR, were determined in the ileum of patients by qPCR and/or Western blot. The *GRP78* expression in R-NEC patients was higher than that in A-NEC-XBP1u patients. Interestingly, A-NEC-XBP1s patients expressed more GRP78 than A-NEC-XBP1u patients (Figure 1B). Similarly, CHOP mRNA expression levels in A-NEC-XBP1s patients were higher compared with A-NEC-XBP1u patients (Figure 1C). Western blot analysis also revealed increased GRP78 and CHOP protein expression in A-NEC-XBP1s patients compared with A-NEC-XBP1u patients (Figure 1D). 

### Intestinal morphology and localization of GRP78

**Figure 2:**
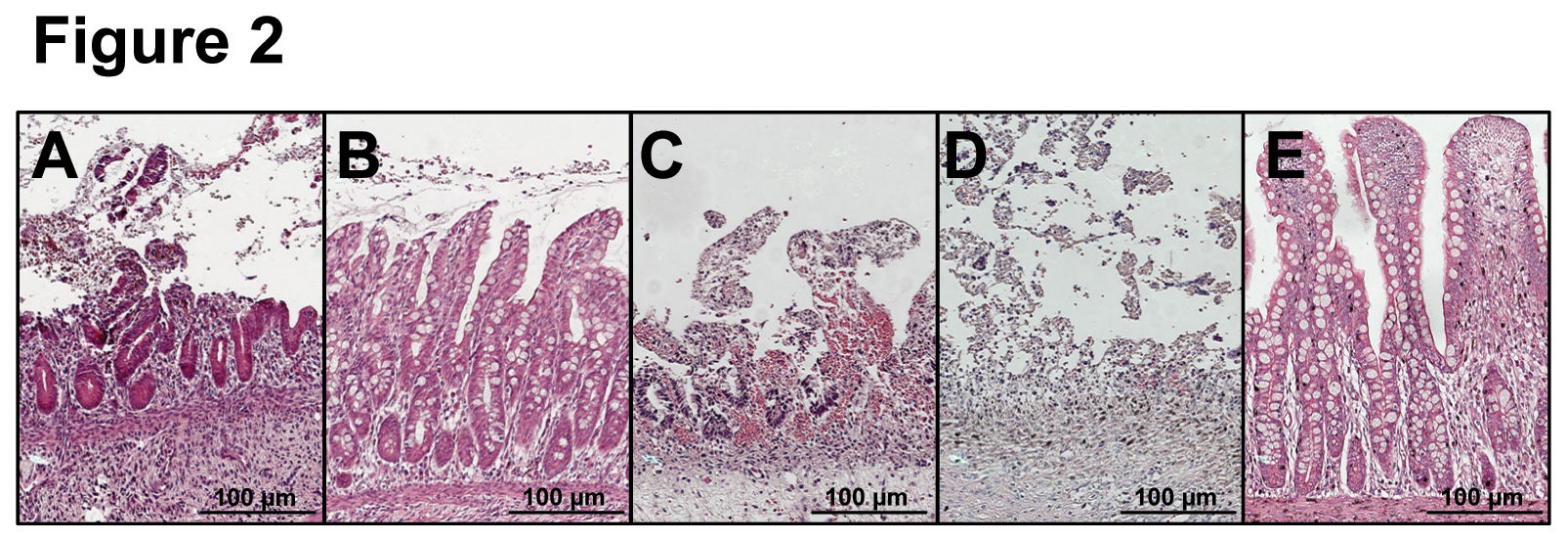
Intestinal morphology of NEC patients. Ileal tissues from NEC patients were stained with hematoxylin and eosin and representative sections are shown. (A) Ileum of an A-NEC-XBP1u patient with severe morphological damage. (B) Ileum of an A-NEC-XBP1u patient with mild morphological damage. (C) Ileum of an A-NEC-XBP1s patient with complete crypt-villus loss but remaining surface epithelium. (D) Ileum of an A-NEC-XBP1s patient who hardly had epithelium left. (E) Ileum of an R-NEC patient with mild mucosal damage.

In line with our previous study [18], most (7 out of 12) of A-NEC patients showed severe morphological damage characterized by crypt-villus loss and/or almost complete villus atrophy, i.e., crypt-villus ratio 1:1 (Figure 2A). Only 5 out of 12 A-NEC patients showed mild damage with relatively normal crypts and villi (Figure 2B). Focusing on A-NEC patients, 2 out of 4 A-NEC-XBP1s patients showed complete crypt-villus loss but a remaining surface epithelium (Figure 2C), and 1 A-NEC-XBP1s patient hardly had epithelium left (Figure 2D). In R-NEC patients, epithelial morphology was restored compared with A-NEC patients, and only mild mucosal damage was observed (Figure 2E). 

GRP78 positive (GRP78^+^) cells were observed in the submucosa, lamina propria, and epithelium of the ileum of all patients (Figure S3A and S3B). GRP78^+^ cells were also found at the base of crypts in most of R-CTRL and R-NEC patients (Figure S3B). In contrast, most of A-CTRL and A-NEC patients did not have GRP78^+^ cells at the base of crypts (data not shown). The position and morphology of these GRP78^+^ cells suggest that these cells are most likely Paneth cells. Therefore, we performed double-stainings for HD5, a specific Paneth cell marker, and GRP78. The GRP78^+^ cells at the base of crypts were also positive for HD5 (Figure S3C), indicating that these cells are indeed Paneth cells. 

### IL6 and IL8 expression levels in NEC patients

**Figure 3:**
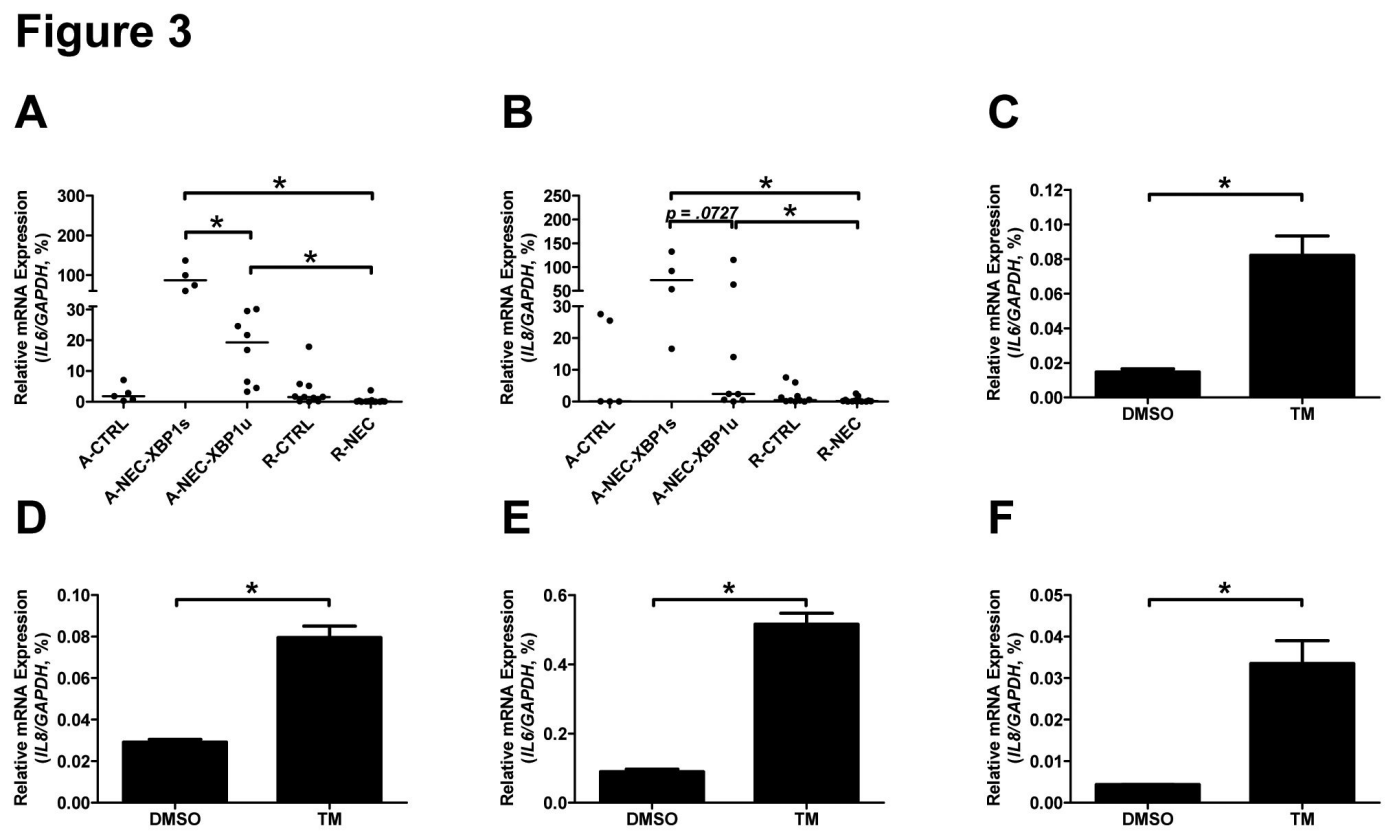
ER stress and the UPR up-regulated the expression levels of *IL6* and *IL8*. The mRNA expression levels of *IL6* (A,C & E) and *IL8* (B, D & F) in the ileum of patients (A&B), THP-1 cells (C & D) and HIEC cells (E & F) were quantified using qPCR and normalized to the mRNA expression levels of *GAPDH*. Asterisks indicate statistical significant differences between indicated groups.

Gene expression levels of *IL6* and *IL8* were evaluated using qPCR. Mucosal *IL6* mRNA expression was increased in A-NEC-XBP1s patients when compared with A-NEC-XBP1u patients, and both A-NEC-XBP1s and A-NEC-XBP1u patients showed higher *IL6* mRNA expression than R-NEC patients (Figure 3A). There was a trend towards increased *IL8* mRNA expression in A-NEC-XBP1s patients compared with A-NEC-XBP1u patients, and both A-NEC-XBP1s and A-NEC-XBP1u patients showed higher *IL8* mRNA expression than R-NEC patients (Figure 3B). 

To analyze whether ER stress and the UPR can increase *IL6* and *IL8* expression directly, THP-1 cells and HIEC cells were treated with TM, an inducer of ER stress and the UPR. In both cell lines, TM-treatment induced ER stress and the UPR characterized by the induction of *XBP1s* (Figure 1A and Figure S4A) and up-regulation of GRP78 (Figure S4B to S4D). Importantly, TM-treatment indeed significantly up-regulated the mRNA expression levels of *IL6* and *IL8* in both cell lines (Figure 3C to 3F), which is similar to what was observed in A-NEC-XBP1s patients. 

### Altered cluster of differentiation 4 positive (CD4^+^) T cells differentiation in NEC patients

**Figure 4:**
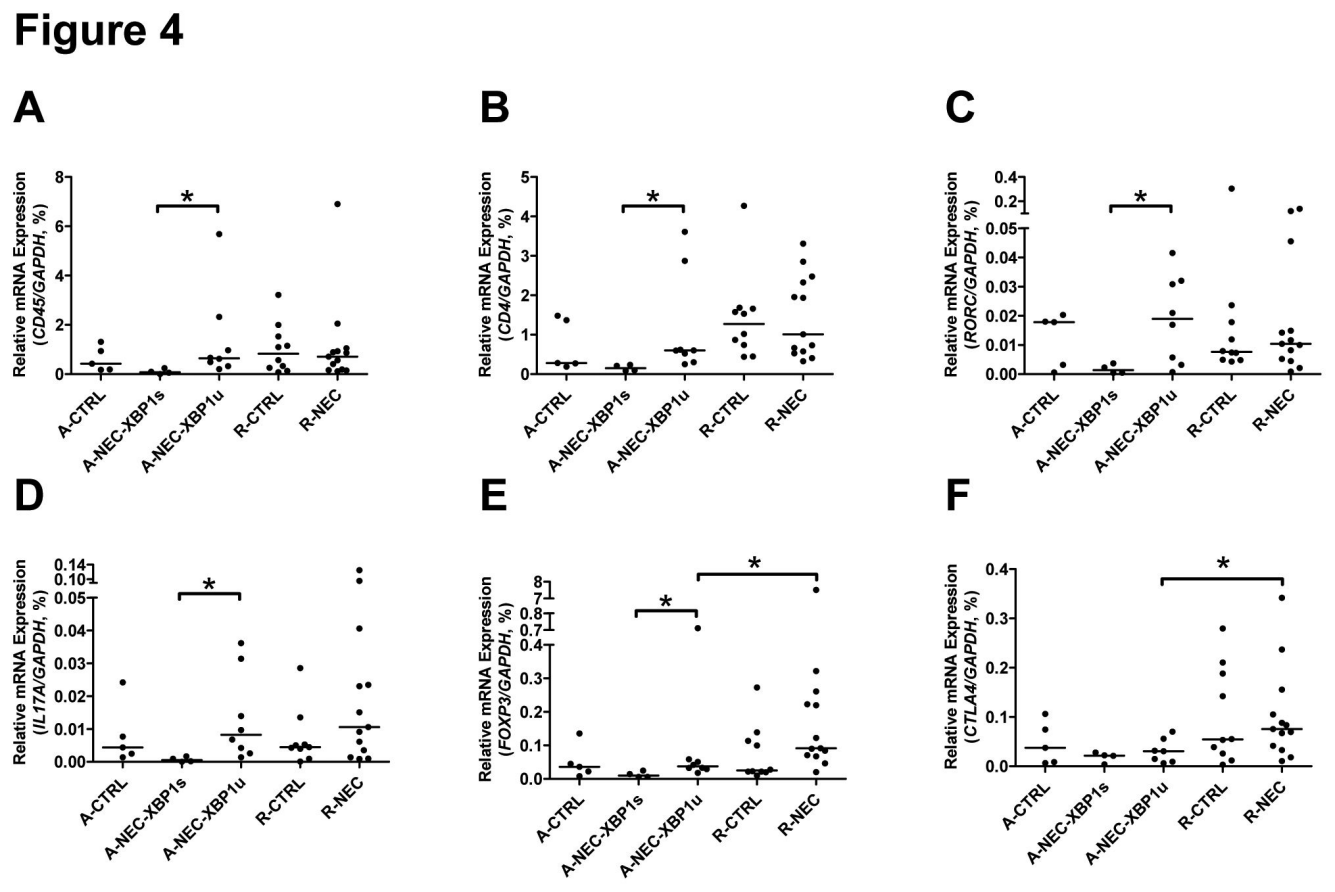
Altered CD4^+^ cells differentiation in NEC patients. Mucosal mRNA expression levels of *CD45* (A), *CD4* (B), *RORC* (C), *IL17A* (D), *FOXP3* (E) and *CTLA4* (F) in the ileum of patients were quantified using qPCR and normalized to *GAPDH* mRNA levels. Asterisks indicate statistical significant differences between indicated groups.

We determined the gene expression of hematopoietic cells (except erythrocytes and plasma/B-cells) and CD4^+^ T cells in A-NEC patients using cluster of differentiation 45 (CD45) and CD4 as markers, respectively. Mucosal mRNA expression levels of *CD45* and *CD4* were significantly decreased in A-NEC-XBP1s patients compared with A-NEC-XBP1u patients when normalized to *GAPDH* (Figure 4A and 4B), suggesting that overall there were less hematopoietic cells and CD4^+^ T cells in A-NEC-XBP1s patients. However, there were no significant differences in *CD4* expression levels when normalized to *CD45* (Figure S5A), implying that the numbers of CD4^+^ T cells relative to hematopoietic cells were not altered between A-NEC-XBP1s and A-NEC-XBP1u patients. 

Since there was an overall decrease in *CD4* expression levels in A-NEC-XBP1s patients compared with A-NEC-XBP1u patients, we analyzed the expression of helper T (Th1, Th2 and Th17) cell markers and regulatory T (Treg) cell markers using *GAPDH* as internal control. Mucosal mRNA expression levels of T-box 21 (*TBX21*) and GATA binding protein 3 (*GATA3*) did not differ between A-NEC-XBP1s and A-NEC-XBP1u patients (Figure S5B and S5C), indicating that the total abundances of Th1 and Th2 cells were not changed between A-NEC-XBP1s and A-NEC-XBP1u patients. The mRNA expression levels of RAR-related orphan receptor C (RORC) and interleukin 17A (*IL17A*) were down-regulated in A-NEC-XBP1s patients compared with A-NEC-XBP1u patients (Figure 4C and 4D), demonstrating that there were less Th17 cells in A-NEC-XBP1s patients. Similarly, mRNA expression levels of *FOXP3*, a hallmark of immune-suppressive Treg cells, were down-regulated in A-NEC-XBP1s patients compared with A-NEC-XBP1u patients (Figure 4E), suggesting reduced Treg cell numbers in A-NEC-XBP1s patients. Interestingly, the mRNA expression levels of *FOXP3* and cytotoxic T-lymphocyte antigen 4 (CTLA4) were up-regulated in R-NEC patients compared with A-NEC-XBP1u patients (Figure 4E and 4F), implying an enhanced immunosuppressive function in R-NEC patients. 

We also identified FOXP3 positive (FOXP3^+^) cells by immunohistochemistry. FOXP3^+^ cells were detected in 6 out of 12 A-NEC patients and all R-NEC patients, and there was a trend towards increased abundance of FOXP3^+^ cells in R-NEC patients compared with A-NEC patients (data not shown). In A-NEC patients, FOXP3^+^ cells were confined to the lymph nodes, submucosa and lamina propria in the crypt region (Figure S6A). In R-NEC patients, besides the lymph nodes, submucosa and lamina propria in the crypt region, FOXP3^+^ cells also localized to lamina propria within the villi, which correlated with areas containing lymphocytic infiltrates. Remarkably, in some R-NEC patients FOXP3^+^ cells were associated with, and interspersed between, epithelial cells (Figure S6B). 

**Figure 5:**
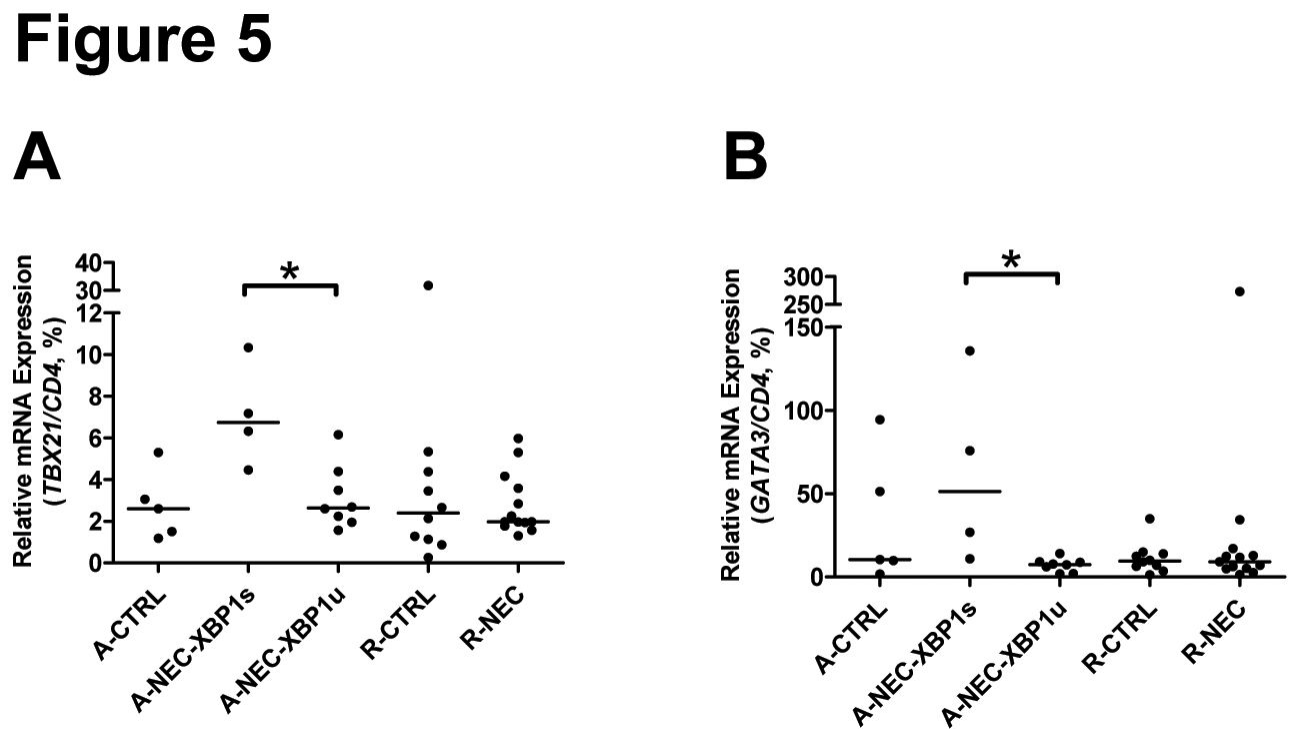
Altered relative amount of CD4^+^ cells in NEC patients. Mucosal mRNA expression levels of *TBX21* (G) and *GATA3* (H) in the ileum of patients were quantified using qPCR and normalized to the mRNA expression levels of *CD4*. Asterisks indicate statistical significant differences between indicated groups.

Finally we normalized the expression of Th cell markers and Treg cells to *CD4* mRNA to determine the relative amount of these cells compared with CD4^+^ cells. In A-NEC-XPB1s patients compared with A-NEC-XBP1u patients, the relative expression levels of *TBX21* and *GATA3* were up-regulated (Figure 5A and 5B), while the relative expression of *RORC*, *IL17A* and *FOXP3* remained unaltered (Figure S5D to S5F). These data suggest that more CD4^+^ T cells had differentiated into a Th1 or Th2 cell than into a Th17 or Treg cell in the A-NEC-XPB1s patients. 

## Discussion

ER stress and the UPR are involved in various chronic inflammatory diseases, including inflammatory bowel disease. However, the role of ER stress and the UPR in NEC, an acute inflammatory disease in premature infants, was unknown. In the present study, we first analyzed ER stress and the UPR in NEC patients, and we determined *IL6* and *IL8* expression and the differentiation of CD4^+^ T cells. Our data indicate that acute NEC patients with ER stress and activation of the UPR show increased *IL6* and *IL8* expression with altered T cell differentiation. 

There are three main pathways (IRE1, PERK and ATF6) sensing ER stress. Splicing of *XBP1* mRNA, which is activated by ER stress via IRE1 pathway, is associated with intestinal inflammation [5]. The spliced form *XBP1s* is a transcriptional activator of UPR target genes; whereas the unspliced form *XBP1u* is a transcriptional suppressor of UPR target genes [19]. *XBP1s* was detected in 1 out of 5 A-CTRL patients and in 4 out of 12 A-NEC patients, but not in R-CTRL and in R-NEC patients. Two general ER stress and UPR markers, GRP78 and CHOP, were both up-regulated in A-NEC-XBP1s patients compared with A-NEC-XBP1u and R-NEC patients, which confirmed the occurrence of ER stress and the UPR in A-NEC-XBP1s patients. Analysis of PERK and ATF6 pathways revealed that the expression levels of *ATF4*, *GADD34* and *PDIA4* were similar between the patient groups studied. With respect to these data, we suggest that PERK and ATF6 pathways were not activated, likely suggesting that only the IRE1 pathway was activated in A-NEC-XBP1s patients. These data indicate that ER stress and activation of the UPR plays a role in NEC, as ER stress and the UPR was observed in a subset of A-NEC patients, but might not occur in all NEC patients. A reason for this might be the complexity of this devastating disease. NEC can be caused by one or a combination of risk factors, i.e., bacterial colonization, formula nutrition and ischemia [20,21]. Because of this, one can consider NEC as a multi-factorial disease, most likely leading to differences in the pathogenesis. The occurrence of ER stress and the UPR in only a subset of A-NEC patients supports the multifactoriality of NEC. 

We observed increased *GRP78* expression levels and increased GRP78^+^ Paneth cell abundances in R-NEC patients compared with A-NEC-XBP1u patients. Paneth cells are among the most highly secretory cell types in mammals, and these cells synthesize large amount of secretory proteins including anti-microbial peptides [22]. To cope with the high basal protein synthesis level, Paneth cells express high basal levels of ER chaperones including GRP78. Studies report that the Paneth cell number is lower in the preterm infants compared with term infants [23,24]. This is in line with our results showing reduced HD5 positive and GRP78^+^ Paneth cells in A-CTRL and A-NEC patients. Paneth cells play a pivotal role in the innate immune response and host defense by producing anti-microbial peptides that shape the gut microbiota [25], and Paneth cell dysfunction has been suggested in NEC [26]. In a previous study we already demonstrated Paneth cell hyperplasia after recovery from NEC [27]. Therefore, the increased *GRP78* expression in R-NEC patients might be due to up-regulated Paneth cell activity, i.e., increased protein synthesis, but this aspect needs more investigation. 

There is growing evidence showing that *IL6* and *IL8* are involved in the pathogenesis of NEC [28]. In the present study, we detected increased mucosal *IL6* and *IL8* expression in A-NEC patients compared with R-NEC patients. Several studies suggest that plasma IL6 concentrations reflect the clinical severity of NEC [29–32]. Plasma IL8 levels were elevated in infants with NEC compared with those with sepsis [33], and it was also higher in infants with stage 3 NEC compared with those with less severe NEC [34]. Our results are in line with the previous studies analyzing IL6 and IL8 levels in plasma of NEC patients and imply an essential role for mucosal IL6 and IL8 in the pathogenesis of NEC. Interestingly, A-NEC-XBP1s patients showed increased *IL6* expression and a trend towards increased *IL8* expression compared with A-NEC-XBP1u patients. It has been reported that ER stress activated NF-κB [35], and XBP1s induces the production of IL6 and IL8 in various cell types [36–38]. Moreover, a positive correlation between *GRP78* and *IL8* expression in the intestine of IBD patients has also been observed [6]. Using the human monocytic cell line THP-1 and the fetal human intestine epithelial cell line HIEC, we confirmed the correlation between ER stress and increased *IL6* and *IL8* expression *in vitro*. Specifically, TM induced GRP78 expression, splicing of *XBP1*, and up-regulated the expression of *IL6* and *IL8* mRNAs in both cell lines. 

Naive CD4^+^ T cells may differentiate into Th cells and Treg cells with their distinct cytokine profiles and immune functions [39]. Treg cells can suppress the effector functions of other types of T cells, and therefore are essential for the immune homeostasis [40]. Recently, reduced numbers of Treg cells were reported in NEC ileum compared with infants without NEC [41]. We did not find differences in *FOXP3* and *CTLA4* gene expression levels between age-matched A-CTRL and A-NEC patients or between R-CTRL and R-NEC patients, but we noticed that R-NEC patients expressed higher *FOXP3* and *CTLA4* levels than both A-NEC-XBP1u and A-NEC-XBP1s patients. Accordingly, compared with A-NEC patients, there was a trend towards to increased FOXP3^+^ cell in the ileum of R-NEC patients, and FOXP3^+^ cells appeared to be associated with epithelial cells of R-NEC patients exclusively, suggesting altered Treg cell differentiation and decreased immunosuppressive function in A-NEC patients. However, detailed functional studies are needed to further confirm the important role of Treg in NEC recovery since FOXP3 can also be induced in activated T effector cells [42].

Detailed analysis reveals that there might be a link between ER stress and altered CD4^+^ cells differentiation as seen in A-NEC-XBP1s patients. Total *CD4* gene expression levels were lower in A-NEC-XBP1s patients than in A-NEC-XBP1u patients. Using the key transcription factors as T cell markers, we demonstrate that the total abundance of Th1 and Th2 cells remained unaltered, while the total abundance of Th17 cells and Treg cells were decreased in A-NEC-XBP1s patients compared with A-NEC-XBP1u patients. These data imply that decreased abundances in Th17 and Treg cells are the cause of the decrease in total abundance of CD4^+^ T cells. Accordingly, the relative amounts of Th1 and Th2 cells were higher in A-NEC-XBP1s patients when *TBX21* and *GATA3* were normalized to *CD4*. We also confirmed the altered Th17 cell differentiation using the signature cytokine IL17A. ER stress and the UPR can regulate Th17 cells, as overexpression of CHOP in developing Th17 cells suppresses IL17A production [43]. Th17 cells and IL17A can directly inhibit Th1 cells, and thereby suppress the development of intestinal inflammation [44]. Interestingly, it has been shown that the Th17-cell capacity is inversely related to developmental age and that preterm infants show a significant Th17-cell bias [45]. Therefore, it can be hypothesized that ER stress inhibits the differentiation of Th17 cells in A-NEC-XBP1s patients, whom were born prematurely, resulting in more severe inflammation and necrosis. Yet, further studies are necessary to confirm this hypothesis. Nevertheless, all data in conjunction point to an imbalance in CD4^+^ T cells differentiation in A-NEC-XBP1s patients. Additionally, changes in 

RORC, IL17A and FOXP3 gene expression levels might also reflect a dysregulation of a group of innate immune cells called innate lymphoid cells. However, the exact role of these innate lymphoid cells in NEC needs further investigation.

*XBP1s* correlated with a poor epithelial morphology and surgical outcome. Two of 4 A-NEC-XBP1s patients showed complete crypt and villus loss, and 1 A-NEC-XBP1s patient only had limited epithelium left. Three of the 4 A-NEC-XBP1s patients died within 1 year after surgery. In contrast, A-NEC-XBP1u patients had relatively better epithelial morphology than A-NEC-XBP1s patients and surgical outcome was also better in A-NEC-XBP1u patients as 3 out of 8 A-NEC-XBP1u patients died within 1 year after surgery compared with 3 out of 4 A-NEC-XBP1s patients. However, there was no significant difference between the survival rates of A-NEC-XBP1s and A-NEC-XBP1u patients (25.0% versus 62.5%), probably due to the small patient numbers. Finally, in most R-NEC patients, the epithelial morphology was restored, and only 1 out of 13 R-NEC patients died within 1 year after surgery. 

In summary, ER stress and the UPR correlate with the disease severity in a subset of NEC patients. Compared with A-NEC-XBP1u patients, A-NEC-XBP1s patients suffer from more severe inflammation, have increased ER stress and activated UPR, as demonstrated by *XBP1s* and increased GRP78 and CHOP expression, and have a worse surgical outcome. Accordingly, A-NEC-XBP1s patients express higher levels of *IL6*, and show a trend towards increased *IL8* expression compared with A-NEC-XBP1u patients. The total abundance of CD4^+^ T cells is reduced in A-NEC-XBP1s patients compared with A-NEC-XBP1u patients, and T cells differentiation is also altered. Specifically, in A-NEC-XBP1s patients, Th17 and Treg functions seem suppressed and the relative amounts of Th1 and Th2 cells seem up-regulated in A-NEC-XBP1s patients. In line with this, the mucosal damage is more severe in A-NEC-XBP1s patients compared with A-NEC-XBP1u patients. Furthermore, R-NEC patients did not show splicing of *XBP1* mRNA. In R-NEC patients, *IL6* and *IL8* are suppressed, Treg cells are up-regulated and epithelial morphology is restored. Together, these data imply that that prevention/avoidance of ER stress and the UPR in NEC patients may contribute to improved disease outcome.

## Supporting Information

Figure S1 **Three pathways of ER stress and the UPR.** IRE1 functions as an endoribonuclease and protein kinase. Upon activation, IRE1 removes a 26-bp nucleotide fragment from cytosolic unspliced XBP1 mRNA to generate spliced XBP1 which encodes a transcription factor inducing UPR target genes.
During ER stress, PERK is autophosphorylated, resulting in phosphorylation of EIF2A. This in turn leads to an arrest in protein translation and accumulation of ATF4. GADD34 is also induced by the PERK pathway and dephosphorylates EIF2A to restores protein translation.
Upon ER stress, ATF6 is cleaved and the active ATF6 p50 subunit moves to the nucleus to modulate gene expression such as PDIA4.
(TIF)

Figure S2 **Expression of genes of PERK and ATF6 pathways in the ileum of patients.** The mucosal mRNA expression levels of *ATF4* (A), *GADD34* (B) and *PDIA4* (C) in the ileum of patients were quantified using qPCR and normalized to the mRNA expression levels of *GAPDH*.
(TIF)

Figure S3 **Localization of GRP78 in the ileum of NEC patients.** The localization of GRP78 in the ileum of NEC patients was investigated using IHC (A & B) and IF (C).
(A) Representative staining of GRP78 in A-NEC patients is shown. Black arrows indicate the GRP78^+^ cells in submucosa (SM), lamina propria (LP) and surface epithelium (SE).
(B) Representative staining of GRP78 in R-NEC patients is shown. Black arrows indicate the GRP78^+^ cells in SM, LP, SE and base of crypts (BC).
(C) Representative IF staining shows the co-localization of GRP78 and HD5 in the Paneth cells in R-NEC patients. Green signals in the top-left panel show the GRP78 staining, red signals in the top-right panel show the HD5 staining, and the bottom-left panel shows the merged signals. White arrows indicate the GRP78 and HD5 co-localized Paneth cells.
(TIF)

Figure S4 **TM-induced ER stress and the UPR *invitro*.** (A) The splicing of *XBP1* induced by TM was detected, and the representative PCR products of *XBP1u* and *XBP1s* in THP-1 cells are shown using DNA electrophoresis.
(B) The induction of GRP78 protein expression by TM in HIEC and THP-1 cells was demonstrated using Western blot.
The mRNA expression levels of *GRP78* in the DMSO-treated and TM-treated THP-1 (C) and HIEC (D) cells were quantified using qPCR and normalized to the mRNA expression levels of *GAPDH*. Asterisks indicate statistical significant differences between DMSO and TM groups.
(TIF)

Figure S5 **Expression of CD4^+^ T cells in the ileum of patients.** Mucosal mRNA expression levels of *CD4* in the ileum of patients were quantified using qPCR and normalized to the mRNA expression levels of *CD45* to demonstrate the amount of CD4^+^ cells in all hematopoietic cells (A). Mucosal mRNA expression levels of *TBX21* (B) and *GATA3* (C) in the ileum of patients were quantified using qPCR and normalized to the mRNA expression levels of *GAPDH*. Mucosal mRNA expression levels of *RORC* (D), *IL17A* (E) and *FOXP3* (F) in the ileum of patients were quantified using qPCR and normalized to the mRNA expression levels of *CD4*.
(TIF)

Figure S6 **Localization of FOXP3^+^ cells in A-NEC and R-NEC patients.** (A) Representative FOXP3 stainings in A-NEC patients is shown. Black arrows indicate the FOXP3^+^ cells in the lymph node (LN), submucosa (SM), and lamina propria in the crypt region (LPC).
(B) Representative FOXP3 stainings in R-NEC patients is shown. Black arrows indicate the FOXP3^+^ cells in the LN, SM, LPC and lamina propria within the villi (LPV). Black triangles indicate the FOXP3^+^ cells which interspersed between epithelial cells (IBE). The bottom-right panel with an asterisk represents a higher magnification of IBE.
(TIF)

## References

[1] Schröder M (2008) Endoplasmic reticulum stress responses. Cell Mol Life Sci 65: 862-894. doi:10.1007/s00018-007-7383-5. PubMed: 18038217. 18038217 PMC11131897

[2] McGuckin MA, Eri RD, Das I, Lourie R, Florin TH (2010) ER stress and the unfolded protein response in intestinal inflammation. Am J Physiol Gastrointest Liver Physiol 298: G820-G832. doi:10.1152/ajpgi.00063.2010. PubMed: 20338921. 20338921

[3] Kaser A, Blumberg RS (2010) Endoplasmic reticulum stress and intestinal inflammation. Mucosal Immunol 3: 11-16. doi:10.1038/mi.2009.122. PubMed: 19865077. 19865077 PMC4592136

[4] Heazlewood CK, Cook MC, Eri R, Price GR, Tauro SB et al. (2008) Aberrant mucin assembly in mice causes endoplasmic reticulum stress and spontaneous inflammation resembling ulcerative colitis. PLOS Med 5: e54. doi:10.1371/journal.pmed.0050054. PubMed: 18318598. 18318598 PMC2270292

[5] Kaser A, Lee AH, Franke A, Glickman JN, Zeissig S et al. (2008) XBP1 links ER stress to intestinal inflammation and confers genetic risk for human inflammatory bowel disease. Cell 134: 743-756. doi:10.1016/j.cell.2008.07.021. PubMed: 18775308. 18775308 PMC2586148

[6] Bogaert S, De Vos M, Olievier K, Peeters H, Elewaut D et al. (2011) Involvement of endoplasmic reticulum stress in inflammatory bowel disease: a different implication for colonic and ileal disease? PLOS ONE 6: e25589. doi:10.1371/journal.pone.0025589. PubMed: 22028783. 22028783 PMC3196494

[7] Barmada MM, Brant SR, Nicolae DL, Achkar JP, Panhuysen CI et al. (2004) A genome scan in 260 inflammatory bowel disease-affected relative pairs. Inflamm Bowel Dis 10: 513-520. doi:10.1097/00054725-200409000-00004. PubMed: 15472510. 15472510

[8] Hampe J, Schreiber S, Shaw SH, Lau KF, Bridger S et al. (1999) A genomewide analysis provides evidence for novel linkages in inflammatory bowel disease in a large European cohort. Am J Hum Genet 64: 808-816. doi:10.1086/302294. PubMed: 10053016. 10053016 PMC1377799

[9] Vermeire S, Rutgeerts P, Van Steen K, Joossens S, Claessens G et al. (2004) Genome wide scan in a Flemish inflammatory bowel disease population: support for the IBD4 locus, population heterogeneity, and epistasis. Gut 53: 980-986. doi:10.1136/gut.2003.034033. PubMed: 15194648. 15194648 PMC1774099

[10] Neu J, Walker WA (2011) Necrotizing enterocolitis. N Engl J Med 364: 255-264. doi:10.1056/NEJMra1005408. PubMed: 21247316. 21247316 PMC3628622

[11] Berman L, Moss RL (2011) Necrotizing enterocolitis: An update. Semin Fetal Neonatal Med 16: 145-150. doi:10.1016/j.siny.2011.02.002. PubMed: 21514258. 21514258

[12] Bell MJ, Ternberg JL, Feigin RD, Keating JP, Marshall R et al. (1978) Neonatal necrotizing enterocolitis. Therapeutic decisions based upon clinical staging. Ann Surg 187: 1-7. doi:10.1097/00000658-197801000-00001. PubMed: 413500. 413500 PMC1396409

[13] Perreault N, Beaulieu JF (1996) Use of the dissociating enzyme thermolysin to generate viable human normal intestinal epithelial cell cultures. Exp Cell Res 224: 354-364. doi:10.1006/excr.1996.0145. PubMed: 8612712. 8612712

[14] Van der Sluis M, De Koning BA, De Bruijn AC, Velcich A, Meijerink JP et al. (2006) Muc2-deficient mice spontaneously develop colitis, indicating that MUC2 is critical for colonic protection. Gastroenterology 131: 117-129. doi:10.1053/j.gastro.2006.04.020. PubMed: 16831596. 16831596

[15] Lu P, Burger-van Paassen N, van der Sluis M, Witte-Bouma J, Kerckaert JP et al. (2011) Colonic gene expression patterns of mucin Muc2 knockout mice reveal various phases in colitis development. Inflamm Bowel Dis 17: 2047-2057. doi:10.1002/ibd.21592. PubMed: 21910166. 21910166

[16] Afonyushkin T, Oskolkova OV, Philippova M, Resink TJ, Erne P et al. (2010) Oxidized phospholipids regulate expression of ATF4 and VEGF in endothelial cells via NRF2-dependent mechanism: novel point of convergence between electrophilic and unfolded protein stress pathways. Arterioscler Thromb Vasc Biol 30: 1007-1013. doi:10.1161/ATVBAHA.110.204354. PubMed: 20185790. 20185790

[17] Ma Y, Hendershot LM (2003) Delineation of a negative feedback regulatory loop that controls protein translation during endoplasmic reticulum stress. J Biol Chem 278: 34864-34873. doi:10.1074/jbc.M301107200. PubMed: 12840028. 12840028

[18] Schaart MW, de Bruijn AC, Bouwman DM, de Krijger RR, van Goudoever JB et al. (2009) Epithelial functions of the residual bowel after surgery for necrotising enterocolitis in human infants. J Pediatr Gastroenterol Nutr 49: 31-41. doi:10.1097/MPG.0b013e318186d341. PubMed: 19458550. 19458550

[19] Yoshida H, Oku M, Suzuki M, Mori K (2006) pXBP1(U) encoded in XBP1 pre-mRNA negatively regulates unfolded protein response activator pXBP1(S) in mammalian ER stress response. J Cell Biol 172: 565-575. doi:10.1083/jcb.200508145. PubMed: 16461360. 16461360 PMC2063676

[20] Guner YS, Franklin AL, Chokshi NK, Castle SL, Pontarelli E et al. (2011) P-glycoprotein induction by breast milk attenuates intestinal inflammation in experimental necrotizing enterocolitis. Lab Invest 91: 1668-1679. doi:10.1038/labinvest.2011.113. PubMed: 21788941. 21788941 PMC3909679

[21] Gregory KE, Deforge CE, Natale KM, Phillips M, Van Marter LJ (2011) Necrotizing enterocolitis in the premature infant: neonatal nursing assessment, disease pathogenesis, and clinical presentation. Adv Neonatal Care 11: 155-165; quiz: 21730907. 21730907 10.1097/ANC.0b013e31821baaf4 PMC3759524

[22] Selsted ME, Ouellette AJ (2005) Mammalian defensins in the antimicrobial immune response. Nat Immunol 6: 551-557. doi:10.1038/ni1206. PubMed: 15908936. 15908936

[23] Mallow EB, Harris A, Salzman N, Russell JP, DeBerardinis RJ et al. (1996) Human enteric defensins. Gene structure and developmental expression. J Biol Chem 271: 4038-4045. doi:10.1074/jbc.271.8.4038. PubMed: 8626737. 8626737

[24] Ouellette AJ, Niklas V (2010 ) Paneth Cells in Intestinal Health and Disease in the Newborn. Neoreviews 11: e551 -e557. doi:10.1542/neo.11-10-e551.

[25] Salzman NH, Underwood MA, Bevins CL (2007) Paneth cells, defensins, and the commensal microbiota: a hypothesis on intimate interplay at the intestinal mucosa. Semin Immunol 19: 70-83. doi:10.1016/j.smim.2007.04.002. PubMed: 17485224. 17485224

[26] Coutinho HB, da Mota HC, Coutinho VB, Robalinho TI, Furtado AF et al. (1998) Absence of lysozyme (muramidase) in the intestinal Paneth cells of newborn infants with necrotising enterocolitis. J Clin Pathol 51: 512-514. doi:10.1136/jcp.51.7.512. PubMed: 9797727. 9797727 PMC500803

[27] Puiman PJ, Burger-Van Paassen N, Schaart MW, De Bruijn AC, De Krijger RR et al. (2011) Paneth cell hyperplasia and metaplasia in necrotizing enterocolitis. Pediatr Res 69: 217-223. doi:10.1203/PDR.0b013e3182092a9a. PubMed: 21372757. 21372757

[28] Markel TA, Crisostomo PR, Wairiuko GM, Pitcher J, Tsai BM et al. (2006) Cytokines in necrotizing enterocolitis. Shock 25: 329-337. doi:10.1097/01.shk.0000192126.33823.87. PubMed: 16670633. 16670633

[29] Morecroft JA, Spitz L, Hamilton PA, Holmes SJ (1994) Plasma cytokine levels in necrotizing enterocolitis. Acta Paediatr Suppl 396: 18-20. PubMed: 8086674. 8086674 10.1111/j.1651-2227.1994.tb13235.x

[30] Harris MC, Costarino AT Jr., Sullivan JS, Dulkerian S, McCawley L et al. (1994) Cytokine elevations in critically ill infants with sepsis and necrotizing enterocolitis. J Pediatr 124: 105-111. doi:10.1016/S0022-3476(94)70264-0. PubMed: 8283358. 8283358

[31] Goepfert AR, Andrews WW, Carlo W, Ramsey PS, Cliver SP et al. (2004) Umbilical cord plasma interleukin-6 concentrations in preterm infants and risk of neonatal morbidity. Am J Obstet Gynecol 191: 1375-1381. doi:10.1016/j.ajog.2004.06.086. PubMed: 15507968. 15507968

[32] Morecroft JA, Spitz L, Hamilton PA, Holmes SJ (1994) Plasma interleukin-6 and tumour necrosis factor levels as predictors of disease severity and outcome in necrotizing enterocolitis. J Pediatr Surg 29: 798-800. doi:10.1016/0022-3468(94)90374-3. PubMed: 8078025. 8078025

[33] Harris MC, D'Angio CT, Gallagher PR, Kaufman D, Evans J et al. (2005) Cytokine elaboration in critically ill infants with bacterial sepsis, necrotizing entercolitis, or sepsis syndrome: correlation with clinical parameters of inflammation and mortality. J Pediatr 147: 462-468. doi:10.1016/j.jpeds.2005.04.037. PubMed: 16227031. 16227031

[34] Edelson MB, Bagwell CE, Rozycki HJ (1999) Circulating pro- and counterinflammatory cytokine levels and severity in necrotizing enterocolitis. Pediatrics 103: 766-771. doi:10.1542/peds.103.4.766. PubMed: 10103300. 10103300

[35] Tam AB, Mercado EL, Hoffmann A, Niwa M (2012) ER stress activates NF-kappaB by integrating functions of basal IKK activity, IRE1 and PERK. PLOS ONE 7: e45078. doi:10.1371/journal.pone.0045078. PubMed: 23110043. 23110043 PMC3482226

[36] Iwakoshi NN, Lee AH, Vallabhajosyula P, Otipoby KL, Rajewsky K et al. (2003) Plasma cell differentiation and the unfolded protein response intersect at the transcription factor XBP-1. Nat Immunol 4: 321-329. doi:10.1038/ni907. PubMed: 12612580. 12612580

[37] Martino ME, Olsen JC, Fulcher NB, Wolfgang MC, O'Neal WK et al. (2009) Airway epithelial inflammation-induced endoplasmic reticulum Ca2+ store expansion is mediated by X-box binding protein-1. J Biol Chem 284: 14904-14913. doi:10.1074/jbc.M809180200. PubMed: 19321437. 19321437 PMC2685672

[38] Ribeiro CM, Boucher RC (2010) Role of endoplasmic reticulum stress in cystic fibrosis-related airway inflammatory responses. Proc Am Thorac Soc 7: 387-394. doi:10.1513/pats.201001-017AW. PubMed: 21030518. 21030518 PMC3136959

[39] Zhu J, Yamane H, Paul WE (2010) Differentiation of effector CD4 T cell populations (*). Annu Rev Immunol 28: 445-489. doi:10.1146/annurev-immunol-030409-101212. PubMed: 20192806. 20192806 PMC3502616

[40] Zenewicz LA, Antov A, Flavell RA (2009) CD4 T-cell differentiation and inflammatory bowel disease. Trends Mol Med 15: 199-207. doi:10.1016/j.molmed.2009.03.002. PubMed: 19362058. 19362058

[41] Weitkamp JH, Koyama T, Rock MT, Correa H, Goettel JA et al. (2012) Necrotising enterocolitis is characterised by disrupted immune regulation and diminished mucosal regulatory (FOXP3)/effector (CD4, CD8) T cell ratios. Gut 62: 73-82. PubMed: 22267598. 22267598 10.1136/gutjnl-2011-301551 PMC3606820

[42] Allan SE, Crome SQ, Crellin NK, Passerini L, Steiner TS et al. (2007) Activation-induced FOXP3 in human T effector cells does not suppress proliferation or cytokine production. Int Immunol 19: 345-354. doi:10.1093/intimm/dxm014. PubMed: 17329235. 17329235

[43] Chang SH, Chung Y, Dong C (2010) Vitamin D suppresses Th17 cytokine production by inducing C/EBP homologous protein (CHOP) expression. J Biol Chem 285: 38751-38755. doi:10.1074/jbc.C110.185777. PubMed: 20974859. 20974859 PMC2998156

[44] O'Connor W Jr., Kamanaka M, Booth CJ, Town T, Nakae S et al. (2009) A protective function for interleukin 17A in T cell-mediated intestinal inflammation. Nat Immunol 10: 603-609. doi:10.1038/ni.1736. PubMed: 19448631. 19448631 PMC2709990

[45] Black A, Bhaumik S, Kirkman RL, Weaver CT, Randolph DA (2012) Developmental regulation of Th17-cell capacity in human neonates. Eur J Immunol 42: 311-319. doi:10.1002/eji.201141847. PubMed: 22101893. 22101893 PMC3414367

