# Supplementary figures and images for: Endoplasmic Reticulum Stress, Unfolded Protein Response and Altered T Cell Differentiation in Necrotizing Enterocolitis
Source: PLoS One. 2013 Oct 23;8(10):e78491. doi: 10.1371/journal.pone.0078491 (PMC3806824; doi:10.1371/journal.pone.0078491)

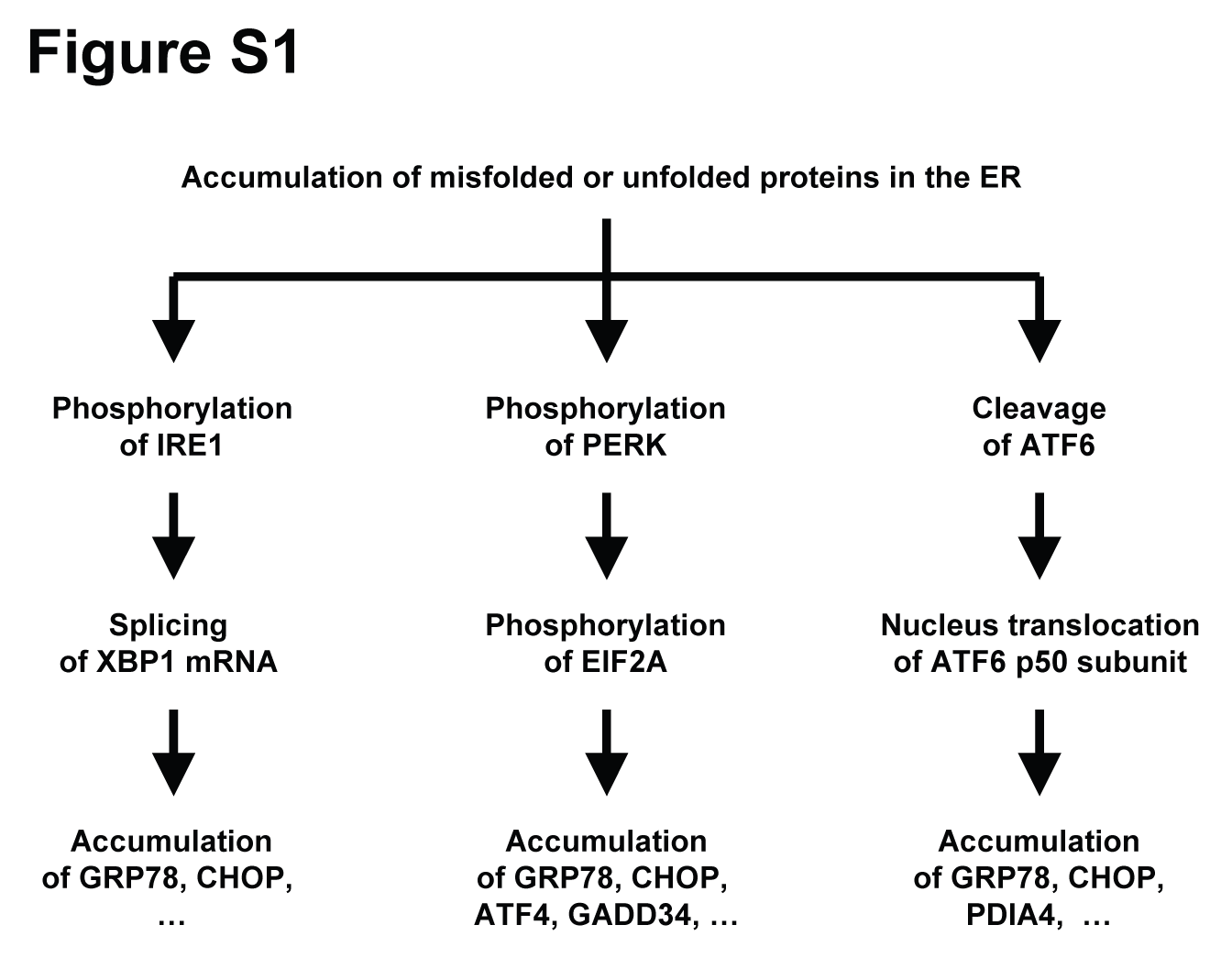

Supplement: Figure S1 — Three pathways of ER stress and the UPR. IRE1 functions as an endoribonuclease and protein kinase. Upon activation, IRE1 removes a 26-bp nucleotide fragment from cytosolic unspliced XBP1 mRNA to generate spliced XBP1 which encodes a transcription factor inducing UPR target genes. During ER stress, PERK is autophosphorylated, resulting in phosphorylation of EIF2A. This in turn leads to an arrest in protein translation and accumulation of ATF4. GADD34 is also induced by the PERK pathway and dephosphorylates EIF2A to restores protein translation. Upon ER stress, ATF6 is cleaved and the active ATF6 p50 subunit moves to the nucleus to modulate gene expression such as PDIA4. (TIF) [file pone.0078491.s001.tif]

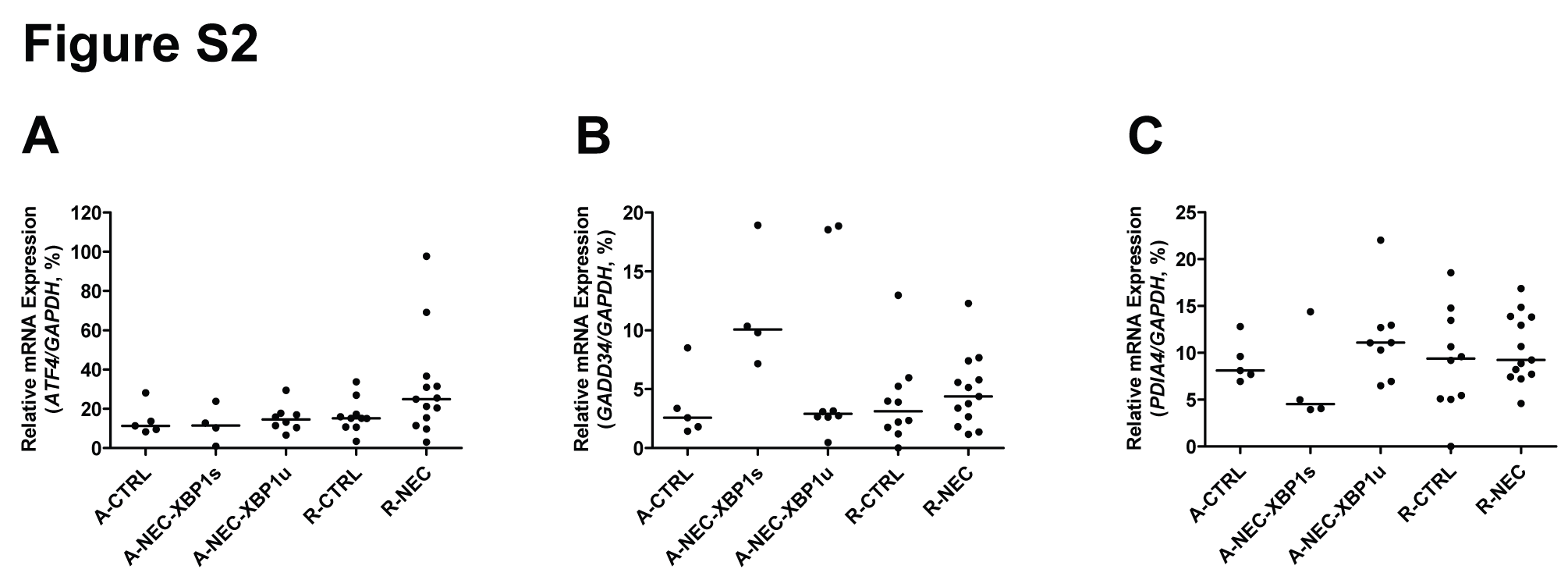

Supplement: Figure S2 — Expression of genes of PERK and ATF6 pathways in the ileum of patients. The mucosal mRNA expression levels of ATF4 (A), GADD34 (B) and PDIA4 (C) in the ileum of patients were quantified using qPCR and normalized to the mRNA expression levels of GAPDH. (TIF) [file pone.0078491.s002.tif]

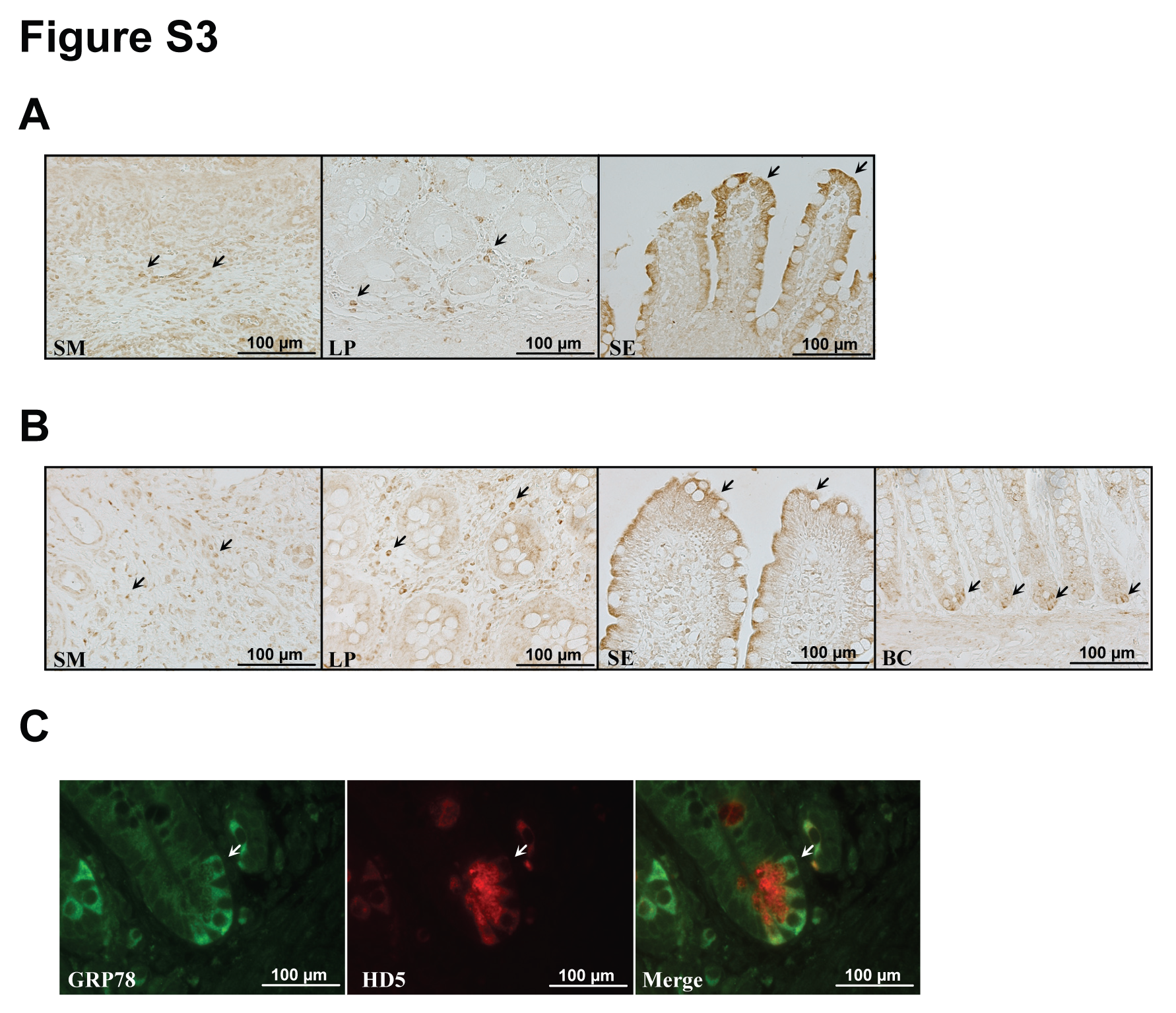

Supplement: Figure S3 — Localization of GRP78 in the ileum of NEC patients. The localization of GRP78 in the ileum of NEC patients was investigated using IHC (A & B) and IF (C). (A) Representative staining of GRP78 in A-NEC patients is shown. Black arrows indicate the GRP78+ cells in submucosa (SM), lamina propria (LP) and surface epithelium (SE). (B) Representative staining of GRP78 in R-NEC patients is shown. Black arrows indicate the GRP78+ cells in SM, LP, SE and base of crypts (BC). (C) Representative IF staining shows the co-localization of GRP78 and HD5 in the Paneth cells in R-NEC patients. Green signals in the top-left panel show the GRP78 staining, red signals in the top-right panel show the HD5 staining, and the bottom-left panel shows the merged signals. White arrows indicate the GRP78 and HD5 co-localized Paneth cells. (TIF) [file pone.0078491.s003.tif]

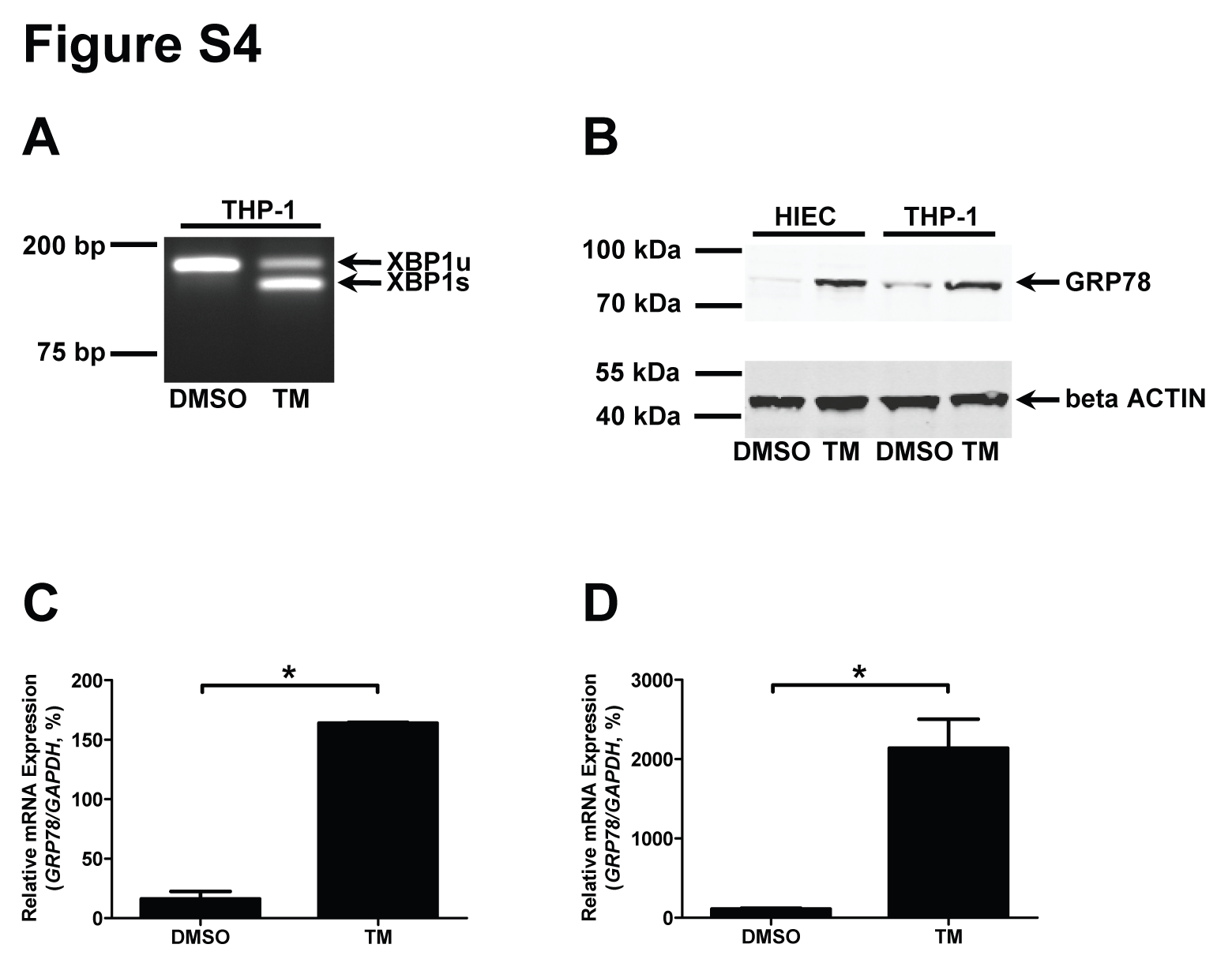

Supplement: Figure S4 — TM-induced ER stress and the UPR invitro. (A) The splicing of XBP1 induced by TM was detected, and the representative PCR products of XBP1u and XBP1s in THP-1 cells are shown using DNA electrophoresis. (B) The induction of GRP78 protein expression by TM in HIEC and THP-1 cells was demonstrated using Western blot. The mRNA expression levels of GRP78 in the DMSO-treated and TM-treated THP-1 (C) and HIEC (D) cells were quantified using qPCR and normalized to the mRNA expression levels of GAPDH. Asterisks indicate statistical significant differences between DMSO and TM groups. (TIF) [file pone.0078491.s004.tif]

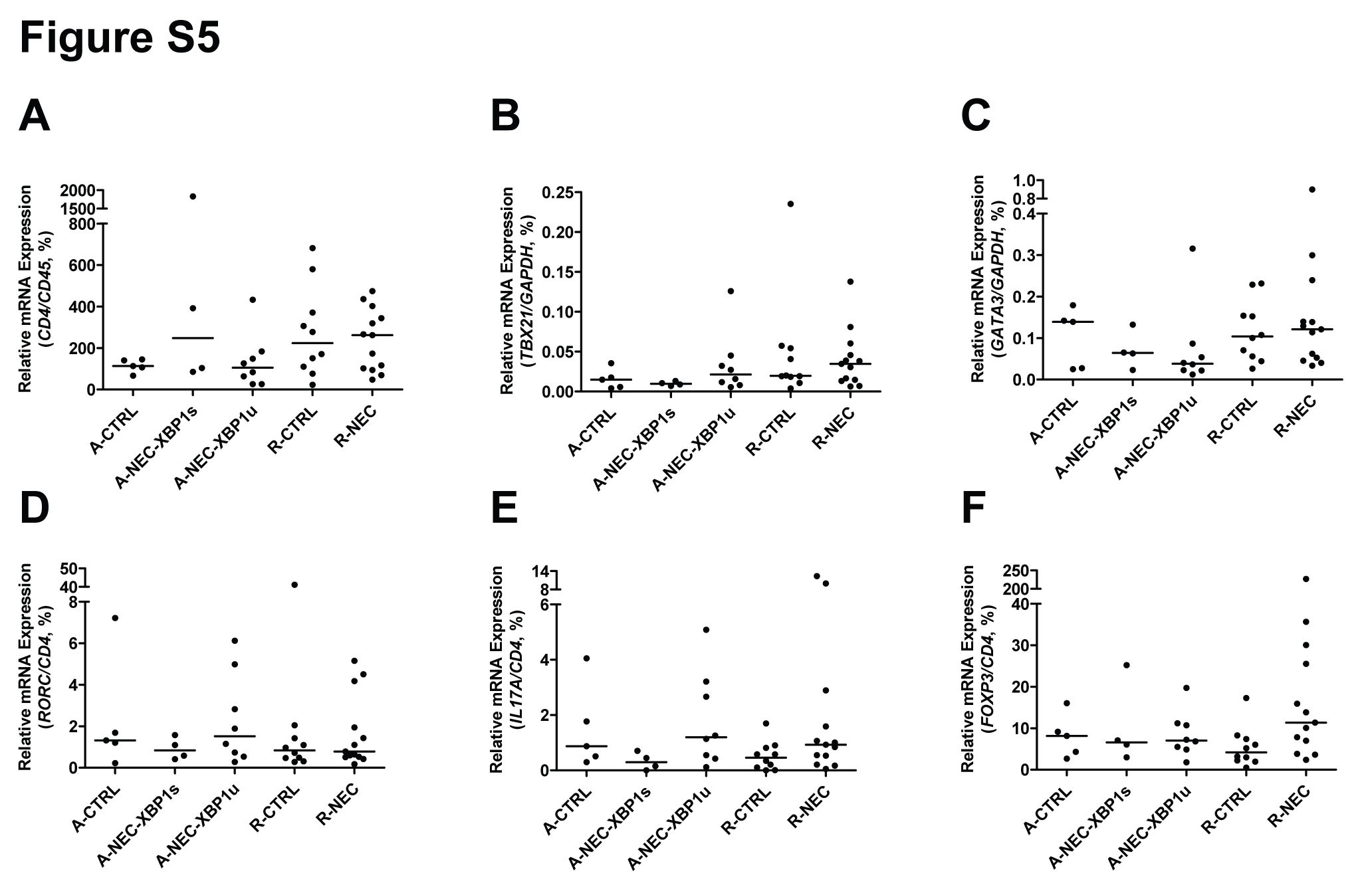

Supplement: Figure S5 — Expression of CD4+ T cells in the ileum of patients. Mucosal mRNA expression levels of CD4 in the ileum of patients were quantified using qPCR and normalized to the mRNA expression levels of CD45 to demonstrate the amount of CD4+ cells in all hematopoietic cells (A). Mucosal mRNA expression levels of TBX21 (B) and GATA3 (C) in the ileum of patients were quantified using qPCR and normalized to the mRNA expression levels of GAPDH. Mucosal mRNA expression levels of RORC (D), IL17A (E) and FOXP3 (F) in the ileum of patients were quantified using qPCR and normalized to the mRNA expression levels of CD4. (TIF) [file pone.0078491.s005.tif]

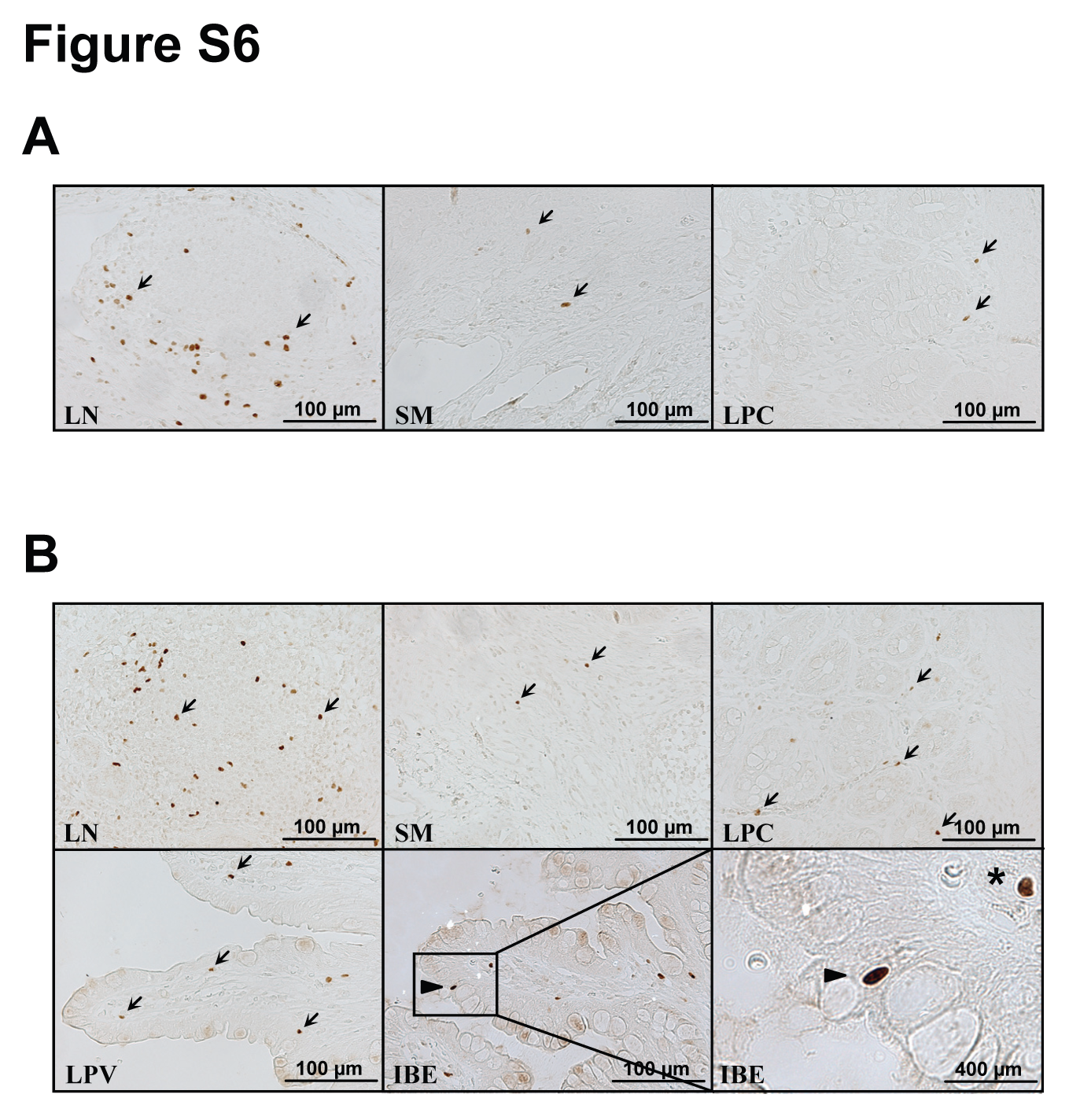

Supplement: Figure S6 — Localization of FOXP3+ cells in A-NEC and R-NEC patients. (A) Representative FOXP3 stainings in A-NEC patients is shown. Black arrows indicate the FOXP3+ cells in the lymph node (LN), submucosa (SM), and lamina propria in the crypt region (LPC). (B) Representative FOXP3 stainings in R-NEC patients is shown. Black arrows indicate the FOXP3+ cells in the LN, SM, LPC and lamina propria within the villi (LPV). Black triangles indicate the FOXP3+ cells which interspersed between epithelial cells (IBE). The bottom-right panel with an asterisk represents a higher magnification of IBE. (TIF) [file pone.0078491.s006.tif]
